# Rapid deployment aortic valve implantation in complex patients with infective endocarditis or aortic valve insufficiency

**DOI:** 10.1186/s13019-024-02967-6

**Published:** 2024-07-16

**Authors:** Kálmán Benke, Viktor Bánhegyi, Edina Korca, Gábor Veres, Yuliana Yakobus, Meradjoddin Matin, Gábor Szabó

**Affiliations:** 1 Heart and Vascular Center, Semmelweis University, Budapest, Hungary; 2 Department of Cardiac Surgery, Martin-Luther University Halle-Wittenberg, Halle an der Saale, Germany

**Keywords:** Off-label, Intuity, Aortic valve replacement, Aortic regurgitation, Infective endocarditis

## Abstract

**Background:**

New prosthetic valves and surgical approaches that shorten operation time and improve the outcome of patients with aortic valve (AV) infective endocarditis (IE) and AV insufficiency (AVI) are crucial. The aim of this study was to evaluate the outcome of patients with AV IE or AVI treated with the EDWARDS INTUITY Rapid-Deployment AV prosthesis for this off-label indication.

**Methods:**

This single-centre retrospective study analyzed data from patients who underwent AV replacement with the EDWARDS INTUITY Rapid-Deployment AV prosthesis for AV IE or regurgitation. (*n* = 8 for IE and *n* = 6 for AVI).

**Results:**

Heart-lung machine times were significantly shorter in the AVI group (111.3 ± 20.7 min) compared to the IE group (171.9 ± 52.4 min) (*p* = 0.02). Aortic cross-clamp followed a similar trend (73.7 ± 9.9 min for AVI vs. 113.4 ± 35.6 min for IE) (*p* = 0.02). The length of ICU stay was also shorter in the AVI group (3.8 ± 2.6 days) compared to the IE group (16.9 ± 8.9 days) (*p* = 0.005). Postoperative echocardiography revealed no paravalvular leakage or significant valvular dysfunction in any patient. One patient died postoperatively from aspiration pneumonia.

**Conclusion:**

The INTUITY valve demonstrates as a safe option for complex AV IE and AVI surgery. Further prospective studies with larger patient cohorts are necessary to confirm these findings and explore the long-term benefits of this approach.

## Introduction

Recent advancements in heart surgery have brought forth rapid deployment aortic valves (RDAVs) as a less invasive alternative for aortic valve replacement (AVR). Primarily used for aortic valve stenosis, RDAVs offer advantages like TAVR, such as shorter procedures and better clinical outcomes.

One such RDAV system is the Edwards INTUITY Aortic bioprosthesis, boasting superior hemodynamic performance and significantly reduced cardiopulmonary bypass times compared to conventional AVR techniques. [1]

However, the use of RDAVs for patients with aortic valve (AV) infective endocarditis (IE) or insufficiency remains relatively unexplored. This is mainly due to concerns about their suitability for these conditions. Encouragingly, recent limited studies suggest that RDAV implantation could be a viable option for select patients with AV IE/AVI. [2–5]

RDAVs represent a modern innovation in heart surgery, designed to expedite and potentially minimize the invasiveness of AVR procedures. Key benefits include reduced surgical time, leading to shorter periods on cardiopulmonary bypass and consequently, fewer related risks. Additionally, RDAVs facilitate minimally invasive surgical techniques, resulting in less postoperative pain, shorter hospital stays, and faster recoveries for patients. Notably, the simplified implantation process can enhance overall patient outcomes, particularly for high-risk or elderly individuals. [6]

Despite these advantages, RDAVs come with some limitations. As a relatively new technology, long-term data on their durability and performance is scarce, making a complete assessment of long-term risks and benefits challenging. Additionally, there’s a potential for paravalvular leaks if the valve doesn’t seal properly, which might be more common compared to traditional valves. The advanced technology behind RDAVs also translates to higher costs, potentially impacting healthcare budgets. Furthermore, certain anatomical factors in patients might hinder or preclude the use of RDAVs. Finally, there’s an increased risk of needing a permanent pacemaker post-surgery due to potential interference with the heart’s conduction system. [7]

Our study aimed to analyse the outcomes of patients with AV IE or AVI who received the INTUITY (Edwards Lifesciences, Irvine, California) bioprosthesis as an off-label treatment for these conditions.

## Materials and methods

This retrospective, single-centre study analyzed data from patients who underwent aortic valve replacement (AVR) with the EDWARDS INTUITY valve between 2016 and 2023. Patients included those with infective AV endocarditis (IE, *n* = 8) and AV regurgitation (*n* = 6). All procedures involved additional interventions besides isolated AVR, except for one patient in the IE group. In the IE group, three patients underwent AV and mitral valve (MV) replacement surgery. One patient received additional coronary artery bypass grafting (CABG) along with AV and MV replacement. The remaining two patients received combined AV replacement with either tricuspid valve (TV) replacement or combined AV, TV, and MV replacement. The AV regurgitation group also received additional procedures alongside AV replacement. Three patients received coronary artery bypass grafts (CABG). One patient received additional MV reconstruction while another received MV replacement. The final patient underwent combined MV and TV replacement surgery.

The study analyzed patient characteristics, pre- and postoperative data, comorbidities, complications and 30-day mortality. Continuous variables are expressed as the mean ± standard deviation (SD); categorical variables are expressed as numbers and percentages. Statistical analyses were performed using GraphPad Prism, version 7.0 (GraphPad Software, Inc., San Diego, CA, USA). All patients provided written informed consent for aortic valve surgery. All surgeries were performed through a median sternotomy approach.

## Results

### Baseline characteristics and preoperative and operative data

The preoperative patient characteristics and operative data are summarised in Table 1. Fourteen patients received the INTUITY valve: eight with IE, and six with aortic valve regurgitation (AR). All patients had multiple comorbidities. The Euroscore II Score was higher in the IE group (12.25 ± 10.96) compared to the AR group (4.26 ± 1.82). While six patients in the IE group and four patients in the AR group had coronary artery disease, only four required bypass surgery. Heart-lung machine time and aortic cross-clamp time were both shorter in the AR group compared to the IE group (171.9 ± 52.41 min vs. 111.3 ± 20.26 min and 113.4 ± 35.57 min vs. 73.67 ± 9.97 min, respectively). These differences were statistically significant (*p* = 0.02 for both). Mean implanted valve size was similar in both groups: 23 mm (range 23–25 mm) in the IE group and 23 mm (range 21–23 mm) in the AR group.

**Table 1:** Preoperative patient characteristics and operative data

	IE (*n* = 8)	AR (*n* = 6)
Preoperative characteristics		
Male	3 (37.5%)	1 (16.7%)
Age (years)	79 ± 5.3	76 ± 6.9
BMI (kg/m²)	27.4 ± 5.6	24.0 ± 4.5
LV-EF (%)	54.5 ± 4.50	50.0 ± 10.49
Euroscore II	12.3 ± 10.9	4.3 ± 1.8
DM 2	3 (37.5%)	1 (16.7%)
Dyslipidemia	3 (37.5%)	5 (83.3%)
Arterial hypertension	8 (100%)	6 (100%)
Peripheral artery disease	3 (37.5%)	0 (0%)
Chronic kidney disease	2 (25%)	2 (33.3%)
COPD	2 (25%)	2 (33.3%)
Atrial fibrillation	4 (50%)	3 (50%)
Heart failure	5 (62.5%)	1 (16.67%)
CAD	6 (75%)	4 (66.7%)
**Operative data**		
Heart lung machine time (min)	171.9 ± 52.4	111.3 ± 20.3
Aortic cross-clamp time (min)	113.4 ± 35.6	73.7 ± 9.9
Implanted valve size (mm)	23.7 ± 1.8	22.3 ± 1.6

Categorical variables are presented as n (%), and continuous variables are presented as the mean ± SD

BMI, body mass index; LV-EF, left ventricular ejection fraction; DM 2, diabetes mellitus type 2; COPD, chronic obstructive pulmonary disease; CAD, coronary artery disease; SD, standard deviation

### Patient specific endocarditis data

Descriptive data from Table 2 revealed two seronegative and one atypical case of endocarditis in addition to the acute cases. Gram-positive cocci were the predominant pathogens. Rifampicin-gentamicin-vancomycin therapy was the preferred antibiotic regimen. Intraoperative abscesses were identified in two patients, and one patient required tricuspid valve and ring reconstruction. Postoperative interventions included pacemaker insertion for two patient and revision surgery for pericardial tamponade in another. Retrospective analysis identified one patient death prior to manuscript completion.

**Table 2:** Endocarditis related clinical characteristics

	Patient I.	Patient II.	Patient III.	Patient IV.	Patient V.	Patient VI.	Patient VII.	Patient VIII.
Endocarditis Subtype	Lenta	Acuta	Lenta	Acuta	Acuta	Acuta	Acuta	Lenta
Germs	Seronegative	E. Coli	Seronegative	Staphyl. Epidermidis	Streptoc. Salivarius	Staphyl. Aureus	Streptoc. Bovis	Atypic Germs
Antibiotics	Ampicillin, Flucloxacillin, Gentamycin	Rifampicin, Gentamycin, Vancomycin	Rifampicin, Gentamycin, Vancomycin, Meropenem	Rifampicin, Gentamycin, Vancomycin	Rifampicin, Gentamycin, Vancomycin	Rifampicin, Gentamycin, Vancomycin	Rifampicin, Gentamycin, Vancomycin	Piperacillin/ Tazobactam, Gentamycin, Vancomycin
Perivalvular abscess	Ø	Ø	Ø	Ø	Ø	Yes	Ø	Yes
Annular reconstruction	Tricuspid- and ring	Ø	Ø	Ø	Ø	Ø	Ø	Ø
Additional interventions	Pacemaker	Tamponade	Pacemaker	ECMO	Tamponade	Ø	Ø	Ø

### Postoperative outcomes

Postoperative patient characteristics are summarised in Table 3. Length of ICU stay was significantly shorter in the aortic regurgitation group (3.83 ± 2.64 days) compared to the infective endocarditis (IE) group (16.88 ± 8.94 days) (*p* = 0.005). Postoperative transoesophageal echocardiography revealed neither paravalvular leakage nor significant valvular dysfunction in any patients. Two patients in the IE group developed postoperative acute kidney injury requiring temporary dialysis. Pacemaker implantation was necessary for two patients in the IE group. and one patient in the AV regurgitation group. Additionally, two patients in the IE group underwent surgical re-exploration for pericardial effusion. Notably, one patient in the AV regurgitation group died postoperatively from aspiration pneumonia, a non-cardiac complication.

**Table 3:** Postoperative patient characteristics

	IE (*n* = 8)	AR (*n* = 6)
Valve leakage	0 (0%)	0 (0%)
Acute renal failure	2 (25%)	0 (0%)
Stroke	1 (12.5%)	0 (0%)
ICU stay (days)	16.9 ± 8.9	3.8 ± 2.6
Hospital stay (days)	27.3 ± 19.1	17 ± 7.2
30-day survival rate	8 (100%)	5 (83.3%)
Pacemaker implantation	2 (25%)	1 (16.7%)
Postoperative bleeding (< 24 h)	2 (25%)	0 (0%)

Categorical variables are expressed as n (%), and continuous variables are expressed as the mean ± SD

## Discussion

The growing number of people requiring aortic valve intervention necessitated the development of new surgical approaches to improve outcomes and prognosis. A significant advance in this area was the introduction of the suturless and RDAV systems approximately 15 years ago.

Several studies suggest improved outcomes for patients treated with these systems, possibly due to shorter cardiopulmonary bypass and aortic clamping durations. One study comparing isolated AVR with sutureless RDAV to conventional AVR reported lower rates of postoperative cardiogenic shock, aortic regurgitation, and atrial fibrillation in the new-technology group. Notably, in our study, only one IE Patient underwent isolated AVR, with a cardiopulmonary bypass time of 93 min and a clamp time of 52 min. The aforementioned study reported a mean bypass time of 74 min and a clamp time of 49 min [8].

Conventional AVR is associated with lower permanent pacemaker (PM) implantation and stroke rates compared to RDAVs. The increased PM need in RDAV recipients likely from compression or injury to the conduction system. However, our patient population originates from Saxony-Anhalt, Germany, an area with some of the highest morbidity and mortality rates in the country, presenting unique challenges in care delivery. Berretta et al. reported a 10.3% incidence of PM implantation in RDAV patients. Interestingly, the incidence of PM implantation for the sutureless Perceval AV and RDAV Intuity in combined surgeries was similar (11.1% vs. 10.3%). [9]

Studies have identified factors associated with PM implantation after RDAV replacement, including right bundle branch block, atrioventricular block, female sex and larger valve size. [10] In our study, 2 patients from the IE group and one from the AVI group received due to postoperative third-degree atrioventricular block. Herry et al. additionally identified endocarditis as a risk factor for PM implantation after AVR. It’s important to acknowledge the limitations of our study, namely the small sample size and the inconsistent follow-up period.

Increased risk of rhythm disturbances (RDs) is a well-documented complication of infective endocarditis (IE). These RDs can range from benign to life-threatening, significantly impacting patient outcomes. While both transcatheter aortic valve implantation (TAVI) and surgical aortic valve replacement (AVR) involve procedures near the aortic valve, the mechanisms leading to rhythm problems differ substantially.

TAVI procedures, while potentially causing transient arrhythmias during valve deployment, are generally associated with a lower risk of long-term RDs compared to AVR. This is likely due to the minimally invasive nature and modern valve design of TAVI. In contrast, IE significantly increases the risk of rhythm problems. The primary culprit in AVR is the body’s inflammatory response to the infection. IE, on the other hand, can directly damage the valve leaflets and surrounding tissues, disrupting the electrical conduction pathways within the heart and promoting arrhythmias. In severe cases, the infection can infiltrate the myocardium itself, further disrupting electrical conduction and promoting potentially life-threatening arrhythmias (Heart Muscle Meltdown). [11]

Recent research suggests that improved valve implantation techniques and identifying predictors for postoperative conduction abnormalities could significantly reduce PM implantation rates. [12–14]

Pleasingly, our study observed no paravalvular leakage or significant valve insufficiency.

Choosing the optimal suturing technique during conventional aortic valve replacement (AVR) involves balancing speed with potential complications. The continuous suture technique boasts faster operation times, a desirable advantage for both surgeons and patients. However, this speed may come at the expense of an increased risk of paravalvular leakage (PVL). PVL occurs when blood leaks around the newly implanted valve, potentially compromising its effectiveness and requiring further intervention. To support this concern, a study comparing continuous and interrupted suturing techniques in bioprosthetic AVR demonstrated a higher rate of PVL with the continuous approach. This finding suggests that surgeons may need to carefully weigh the benefit of faster surgery against the potential drawback of increased leakage when selecting the most suitable suturing technique for each individual patient undergoing AVR. (18)

Piperata et al. emphasize the safety and efficacy of new RDAVs, particularly in complex scenarios like infective endocarditis (IE). Their review highlights promising outcomes, including low mortality and complication rates, and favorable echocardiographic results. The reduced foreign material usage, increased stability in cases with annular involvement, and avoidance of further damage to the compromised annulus associated with RDAVs suggest potential for improved patient outcomes, especially in complex surgeries. [15]

Yun et al. reported the implantation of the INTUITY valve in patients with various AV conditions, including 3 IE patients AVI 20 patients, with encouraging results (one-year hemodynamics data showed mean pressure gradients of 14.7 ± 5.3 and 10.7 ± 3.6 mm Hg in the 19- and 21-mm valves, respectively). [3]

Another study reported implantation of the INTUITY valve in eight patients, with postoperative echocardiographic controls showing a mean transvalvular gradient of 16.7 ± 3.0 mmHg and one case of paravalvular leakage (2 +). [16]

Successful implantation of the EDWARDS INTUITY valve in patients with prosthetic valve IE has also been reported. Belyaev suggests this procedure may be well-suited for patients with fragile root tissue, extensive infection and root abscesses requiring reconstruction. [17]

It is crucial to recognise that the best valve replacement approach depends on various factors, like the patient anatomy, specific condition, and surgeon experience. Ongoing advancements in surgical techniques and improved valve implantation procedures continuously expand the options available for aortic valve surgery, ultimately aiming to enhance patient outcomes and prognosis.

## Conclusions

The use of RDAV INTUITY valve in patients with infective endocarditis or aortic valve regurgitation appears feasible and safe in our study. We observed no technical complications during implantation in these off-label patient groups. This favorable outcome might be attributed to the valve’s anchoring mechanism within the left ventricular outflow tract (LVOT). However, confirmation of these findings requires prospective multicentre studies with larger patient populations.

## Data Availability

The data presented in this study are available on request from the corresponding author.
